# Hospital and Institutional News

**Published:** 1911-11-11

**Authors:** 


### HOSPITAL AND INSTITUTIONAL NEWS.

### THE VOLUNTARY HOSPITALS ORGANISING IN CO-OPERATION.

As we state in a leading article this week, the voluntary hospitals and the medical profession agree that hospital treatment for insured persons must be included in the National Insurance Bill. At a meeting of the Council of the British Hospitals Association last week after full discussion and the receipt of communications from voluntary hospital managers all over the country, the following resolution was unanimously adopted: "That the British Hospitals Association again affirms its unanimous opinion that the National Insurance Bill is incomplete unless it provides for the hospital treatment which insured persons must have if their medical needs are to be covered by the Bill." This resolution bluntly but accurately states the present position of the voluntary hospital managers and the medical profession are now fully seised of the facts; in each district hospital authorities are combining under local committees, and every member of Parliament will in due course have the facts brought to his notice and be urged to uphold the interests of his constituents by pressing upon the Chancellor of the Exchequer the need for hospital treatment under the Bill. An important meeting of all the voluntary hospitals in Glasgow has been organised to take place on the 10th instant, and it is in contemplation to hold a great meeting of representatives of all the voluntary hospitals throughout the United Kingdom in London at an early date. This organisation of the voluntary hospitals on a co-operative basis should prove momentous for good. It should largely contribute to the speedy solution of the problems which Parliament has to face in regard to the setting up of a perfected health organisation, to include a reformed Poor Law and an adequate system of national insurance.

### SIR SHIRLEY MURPHY'S SUCCESSOR.

The official appointment of the new London County Medical Officer for London has not yet been announced officially, but soon after this issue is in the hands of our readers the result will probably be known. The General Purposes Committee meets on Monday when, it is stated, Dr. Alfred Greenwood, Medical Officer of Health, Blackburn; Dr. W. H. Hamer, Medical Officer (General Purposes) L.C.C.; Dr. W. J. Howarth, County Medical Officer, Kent, and Dr. William Butler, Medical Officer of Health, Willesden, will appear.

### A HOSPITAL CHAIRMAN ON THE VALUE OF SANATORIA.

The value of sanatoria was made the occasion of an interesting discussion at the dinner to Dr. Thomas Clarke, of Liverpool, on his retirement from his work at the Consumption Hospital of that city. Dr. Clarke, who has had thirty-two years of institutional experience, was very guarded in his remarks on the effect of sanatoria on the public health, and no doubt he shares the views of Professor Karl Pearson that we discussed in a leading article last week. A retirement, after all, is a very suitable occasion for a little plain speaking, and Dr. Clarke's opinion on this subject would have had great interest since he is well known as having been first appointed physician to the hospital so far back as 1870 and for having been chairman for ten years of the Port Sanitary and Hospital Committee, "the splendid hospital at Fazakerley," as Dr. C. A. Hill remarked, "being a fitting memorial of his chairmanship." Mr. W. Paul, the Treasurer, referred to Dr. Clarke's work in the early days of phthisical study. In short, if anyone was worth drawing on this subject it was the guest of the evening. Dr. Clarke, however, was discreet to the last.

### THE FIRST RECIPIENT OF A NEW DIPLOMA.

The examination for the Diploma in the Diseases and Hygiene of the Tropics, granted by the Conjoint Boards of the Royal College of Physicians, London, and the Royal College of Surgeons, England, (D.T.M. Eng.) was held last week for the first time. J. Bruce-Bays, M.D., a Student of the London School of Tropical Medicine, has the distinction of being the first to obtain this diploma. Dr. Bruce-Bays is Medical Officer of Health, Grahamstown, South Africa, and visiting Medical Officer, Albany General Hospital.

### THE SEAMEN'S AND MILLER HOSPITALS JOINT SCHEME.

So far as the hospital is concerned, pathology is a factor that is costing more every year. Yet with all this expenditure the pathologist is probably the most inadequately remunerated officer in the employ of the average hospital. A highly skilled and competent pathologist should receive at any rate a salary of £500 per annum, but this is a heavy tax on a hospital of about 100 to 200 beds. But pathology lends itself to co-operation, and already in London there are two instances of a pathologist being appointed to more than one hospital. An arrangement has now been entered into by the Seamen's Hospital at Greenwich and the Miller General Hospital for South-East London, whereby these two institutions have appointed the same pathologist. The work of the two hospitals is done in a laboratory common to both, the expenses of assistants, material, and upkeep being shared *pro rata* according to the number of beds. Again, the National Hospital for the Paralysed and Epileptic has an arrangement with St. George's Hospital, whereby the clinical pathology of the first-mentioned is done in the laboratory of St. George's. At one time it was considered essential that the laboratory should be actually in the hospital, but the establishment of clinical research laboratories, to which many of the smaller hospitals have recourse, has shown that this is not necessary. The great advantage of a common laboratory for a group of hospitals lies in the possibility to command the services of a pathologist of the highest attainments who may thus be adequately paid for his work, besides avoiding the necessity of reduplication of costly apparatus.

### THE NORFOLK AND NORWICH HOSPITAL AND THE EYE INFIRMARY.

Considerable discussion took place at the last quarterly meeting of the Board of Governors of this hospital on the resolution "that any further consideration of amalgamation with the Norfolk and Norwich Eye Infirmary be postponed for twelve months." The chairman of the Board of Management, Mr. R. E. Horsfall, in the course of his speech referred to the prejudicial effect that the Insurance Bill would have on the finances of voluntary hospitals and announced that there had already been a very appreciable falling off that year in subscriptions. He, therefore, urged the meeting to pause before adding to their almost overwhelming liabilities. Other speakers laid stress on the great need for an ophthalmic department in the hospital, and it was suggested that to tie their hands for a whole year would be breaking faith with the Eye Infirmary. More than two years ago it had been shown that the lamentable state of affairs at the Eye Infirmary called for immediate action. Mr. Horsfall, in replying to the criticisms, again referred to what in his mind was the principal governing factor in the case—namely, the possible effect of the National Insurance Bill upon contributions to voluntary hospitals. Eventually an amendment was carried by 18 votes to 12 which accepted the quarterly report with the exception of the above resolution, and expressed the hope that "the Board of Management will allow themselves full liberty to reconsider the situation without delay and the best way of escaping from the present difficulties."

### WHAT CHATSWORTH HOUSE DOES FOR THE HOSPITALS.

The most aristocratic turnstile that has ever clicked out money for the hospitals is probably Chatsworth House. Last year people paid £720 for the pleasure of seeing its treasures, and the local hospitals, by the Duke of Devonshire's custom, benefited accordingly. The fine arts have this advantage over every other sort of commodity, that, though continually objects of consumption, their value and wealth-producing quality remain undiminished. The contents of Chatsworth House are undepreciated through the annual enjoyment of them by a thousand pairs of eyes. Indeed, the more they are enjoyed the greater the revenue they produce. The economics of fine art should be a very dear subject to hospital secretaries in Derbyshire, and Mansfield, and Buxton.

### MR. KERSHAW ON HOSPITAL PROGRESS.

There was a substantial attendance of hospital officers at the first sessional meeting of the Hospital Officers Association. The members were assembled to hear the Presidential address of Mr. Richard Kershaw (Secretary of the Central London Throat and Ear Hospital), and he gave a most interesting and carefully studied essay on "A Retrospect and Prospect of our Voluntary Hospitals." "It (the Association) has made its influence felt," he said, "on hospital committees, learned societies and corporate bodies, and the views of its members have had a direct bearing on hospital policy." Presenting in elaborate detail a statement showing the marvellous growth of the voluntary system, Mr. Kershaw pointed out that in 1800 there were in Great Britain but 154 medical institutes supported entirely by voluntary contributions, of which thirty-one were in London. These were continuing their work and in many cases had become general hospitals, but the number had grown in 1900 to over 1,100, no account being taken in these figures of the large number of Samaritan funds and convalescent homes which many hospitals had established. Mr. Alvey (Secretary Charing Cross Hospital) referred to the address as being full of historical and statistical research. "If," he said, "the voluntary system only represents a small proportion of medical relief throughout the country, it has to its credit the sum total of medical scientific advance, for from it all medical progress has been made." This was a good answer to those who urge State aid on the ground of the number of beds in Poor Law institutions, to say nothing of the hospital spirit, which has been the creation of the voluntary hospitals alone.

### LANTERN SLIDES AT AN ANNUAL MEETING.

We are glad to note that Mr. E. Iliffe, the recently chosen Chairman of the Coventry and Warwickshire Hospital, is making vigorous efforts to secure a record attendance at the coming annual meeting on December 15. Annual meetings are merely as dull as hospital committees choose to make them—by leaving them severely alone. In this case the new ward block provides an interesting subject, and the original idea has been adopted of making lantern slides of the architect's drawings, which will be explained by one of the architects, Mr. F. J. Cox. This is an energetic way of attracting public attention, and we prophesy that this annual meeting at any rate will not be the dull routine, or empty the hall as is far too often the case on these occasions. Mr. Iliffe and Mr. Rudd, the Secretary, deserve commendation for their enterprise.

### THE BRIDGWATER HOSPITAL.

The useful work done at the Bridgwater Hospital has now been carried on for nearly a century, and special efforts are being made not only to celebrate this event by freeing the institution from debt, but to carry out a King Edward VII. memorial scheme by adding a children's wing to the hospital. It is proposed to arrange a carnival and fancy fair in the summer of 1912, the proceeds of which shall be devoted to paying off the debt on the hospital and to open a special building fund for the new wing, which it was hoped might be ready to mark the centenary of the institution in 1913. The Bridgwater Hospital, or as it used to be called, Infirmary, is an institution which every effort must be made to keep going, for it is one of the most useful types of small country town hospitals. As Mr. R. Y. Foley pointed out, it has been very economically worked, the housekeeping expenses standing at only about 5s. per head per week. Accommodation for children has long been called for, and from the encouraging report of the public meeting held last week we gather that the new memorial wing has excellent prospects of coming into being in time for the centenary celebrations.

### A NEW CHAIRMAN FOR THE SUNDAY FUND.

Sir C. Ernest Tritton has been appointed chairman of the Finance Committee of the Hospital Sunday Fund, in the place of the late Mr. J. Hampton Hale. Mr. Hale's work has received already a tribute from us. His successor in the chairmanship is the head of a large firm of brokers, was created a baronet in 1905, and for several years has served upon the Finance Committee. For fourteen years he represented Lambeth in the Conservative interest. His connection with the Sunday Fund, together with his financial influence, are aptly acknowledged by his appointment to this chairmanship.

### HOSPITAL HISTORY IN EPITOME.

On more than one occasion we have drawn attention to the desirability of every hospital furnishing its friends and well-wishers with a short and readably-written history of the institution, how it came into existence, what it has done, who were connected with it, and any other points which may interest the general reader. We are therefore glad to note that there is a commendable increase of general interest in hospital histories. Three of our great London institutions have already found their historians. Dr. Peachey's excellent compilation regarding St. George's Hospital is now in course of publication; Mr. Morris's history of the London Hospital is almost a model of what such a book should be; and Sir Samuel Wilks and Mr. Bettany's biographical history of Guy's—which, by the way, stands in need of re-editing—approaches the subject from an interestingly different point. There are, however, several other interesting London institutions which demand the attention of the institutional historian. The history of St. Bartholomew's, our oldest hospital, has for some time been in the press, and Mr. O'Donoughue has fine material for a good " story " of Bedlam, while the National Orthopædic, our ophthalmological hospitals, and the French, Italian, and German institutions, all have many points of interest.

### OPERATIONS IN COTTAGE HOSPITALS.

The growth in the number of operations performed in recent years has placed all those cottage hospitals that are more than five years old in a considerable difficulty. The old operation-room that they possess is, as a rule, quite inadequate for modern surgical work, and legacies do not come in the way of cottage hospitals often enough to make reconstruction or rebuilding an easy matter. Trowbridge Cottage Hospital supplies a case in point. Certain theatre improvements have been wanted here for some time, and now, by the very generous gift of £120 from the Trowbridge Co-operative Society, an opportunity occurs for carrying them out. Mr. W. J. Mann, who has held the office of hon. secretary for some years, is to be congratulated on the opportunity now offered to him and to his committee.

### A MINORITY OF THREE.

At the monthly meeting of the Worcester General Infirmary, a letter was read from the British Hospitals Association, enclosing a resolution passed at their last annual conference, asking the Chancellor of the Exchequer to safeguard the interests of the hospitals in framing the Insurance Bill, which, if passed in its present form would seriously affect hospitals all over the country, both in relation to finance and to the medical profession. The Association asked for the Committee's support. It is interesting to learn that this communication evoked considerable discussion, one member actually proposing the rejection of the resolution, which was, however, agreed to by all but three members.

### HOSPITALS AND STREET NOISES.

Mr. J. J. Barron, the secretary-superintendent of the Bradford Royal Infirmary, did good service in bringing to our notice in a recent letter that " the city council many years ago laid wood paving " on the roads alongside the Infirmary with the benevolent effect of reducing the noise. The Leicester Town Council has just made the road bordering the Infirmary a silent one. Institutional staffs all over the country will share in the thanks of Dr. Pope, the senior physician at Leicester, for the council's thoughtfulness, which he expressed at the recent quarterly meeting. A good work would be accomplished if every hospital resident staff or secretary would write to us, where the surface of the roads contiguous to their institution is still paved, so needlessly, with cobbles or stones. By this means such pressure could be brought to bear on local authorities as could make the noise nuisance largely a thing of the past.

### NEW OFFICERS OF THE BRITISH HOSPITALS ASSOCIATION.

Mr. A. William West has been elected chairman of the council of the above Association. The hon. treasurer, Mr. Thies, being away on his well-earned holiday, Mr. Stewart Johnson has been appointed in his place, while Dr. Mackintosh, M.V.O., is again hon. secretary; and last, but not least, Sir William Cobbett has received the compliment of being elected an honorary member of the Association. Next year the Conference of the Association will be held at Birmingham, thanks to the invitation of Mr. Howard Collins on behalf of the chairman and board of the Birmingham General Hospital. Everyone will feel that the officers who have carried the Association to its present high position are well worthy of being retained. Not a few, we hope, will acknowledge the public spirit and personal service which lead to the heavy work of the British Hospitals Association being voluntarily borne by such already busy men.

### TWENTY-FIVE YEARS OF HOSPITAL SERVICE.

By the resignation of Mr. Claude Douglas on the completion of twenty-five years of honorary service the Leicester Infirmary suffers a severe loss. Mr. Douglas qualified M.R.C.S. in 1873 and L.R.C.P. Lond. in 1874, and in 1888 was elected F.R.C.S. Eng. by examination. After having served for several years as one of the Surgeons of the Leicester Provident Dispensary, he was on November 16, 1886, appointed an Honorary Assistant Surgeon to the Leicester Infirmary on the appointment of his friend and colleague Mr. G. C. Franklin, to the full staff. On February 26, 1901, he was elected an Honorary Surgeon to the institution on the resignation of the late Sir Charles H. Marriott.

### THE BUNN MEMORIAL.

Those who came into personal contact with the late Mr. W. G. Bunn, a memoir of whose work as Secretary to the Hospital Saturday Fund appeared in The Hospital of September 30 (page 683), will be glad to contribute to the memorial now being subscribed for. This, we understand, is to take the form of a portrait in oils, which will be hung in the board room of the Fund's offices. A not very large sum is required for this purpose, and the memorial will be all the more satisfactory if it is the result of the gifts of many small subscribers. These and their like are drawn from the classes which Mr. Bunn's work benefited, and in providing for whom he found his ideals.

### POOR LAW MEDICAL SERVICE IN IRELAND.

The report of the Local Government Board (Ireland) contains references to two cases in which medical officers of the above service have been convicted of bribery. In the first case it was shown to the satisfaction of the Board that the successful candidate for a vacancy in the medical officership of the Annacarriga Dispensary District had been guilty of bribery in connection with his election to this post. The Board accordingly declined to sanction the appointment and further intimated that they should similarly decline to approve the employment of this practitioner in the Poor Law medical service for a period of five years. In the other case the medical officer of the Barronstown Dispensary District was reported by the election judges for bribery in connection with the North Louth Parliamentary election. He was accordingly deprived of his appointment and debarred from all similar appointments for a period of seven years. To rake up afresh these discreditable incidents may seem unnecessary, but we feel bound to record our view that the punishments do not seem nearly severe enough to meet the cases.

### THE DILKE MEMORIAL HOSPITAL.

The proposal already noted to recognise by a memorial the services rendered by Sir Charles Dilke to his country has received already active and very encouraging support. The form which the memorial will take is one for which there has long been great need, namely, a free hospital in the Forest of Dean. In the neighbourhood there is a population of 7,000 miners, quarrymen, tinworkers, and railwaymen, and the average number of accidents, slight and serious, amounts to about twenty per week. The distance by hilly roads to the Gloucester Infirmary is too great to take a badly injured man, and it is proposed that in view of Sir Charles's life-long work on behalf of the labouring poor the memorial should take the form of a hospital at the "Speech House." At this spot, where Courts were held of old to administer the forest and mining law of the district, the hospital would be central, and it is roughly estimated that a substantial beginning of the building and equipment can be made with a sum of £3,000. Lord Beauchamp, as Lord-Lieutenant of Gloucestershire, has accepted the chair of the executive committee, and Sir W. Wedderburn is deputy-chairman. It is proposed to form an international council of influential supporters in order to enlist the sympathy of friends in the Colonies, in India, and the United States. As regards the choice of a locality, it seems peculiarly appropriate that such a memorial to their late member should be located among the workers of his own constituency, since it was the unfailing devotion of the Foresters which enabled him to place his services at the command of the poor and suffering in every part of the world. Sir Charles Dilke's services to the nation and the Empire were so varied and so valuable that we hope there will be an immediate and liberal response. Mr. John Cooksey, of Cinderford, Gloucestershire, is the honorary secretary of the fund which is now being raised.

### THE CLOSED WARDS AT CHARING CROSS HOSPITAL.

The David Livingstone memorial, for which a fund of a million shillings is now being collected, will take the form of freeing Charing Cross Hospital from debt and in re-opening the closed wards under the name of the Livingstone Wing. Such a memorial in the centre of London would be a worthy tribute to the memory of the great medical missionary. The Rev. A. W. Oxford, M.D., reports that progress is being made in the collection of this large fund, but it is hoped that more helpers will come forward to take collecting cards.

### HOSPITAL RAILWAY CARRIAGES.

Certain of the through fast trains on the Austrian railways running to the Bohemian watering places will in future be provided with "hospital" compartments. A considerable number of invalids are carried to these health resorts, and the lack of suitable accommodation has often been detrimental to such travellers or deterrent to those who desire to go. These compartments are to be fitted up with a spring bed and a medicine chest containing a few of the most important drugs and appliances for rendering first-aid. There will also be a water supply to each carriage, and arrangements will be made by which patients can be attended by a medical man who will be attached to the train.

### A FRIENDLY SOCIETIES HOSPITAL.

The intermediate or Friendly Societies Hospital which it is proposed to erect at Melbourne is not likely to take form very soon. The British Medical Association of Victoria has transmitted resolutions to the Friendly Societies, which state that the hospital shall be for patients who are ineligible for public hospitals, but who are unable to pay the customary fees, as well as the charges usually made in private hospitals; and that there shall be no medical staff, but that any medical man may send in patients and attend them; that the medical profession shall be adequately represented on the management; that no patient shall be admitted without first producing a certificate of suitability for admission from a medical practitioner, who should, whenever possible, be the patient's usual medical attendant; that there shall be no out-patients; that the same charge of maintenance shall be made to all patients in any one hospital; that the professional fees shall be arranged between the medical attendant and the patient. The Association of Friendly Societies held a meeting which was very largely attended, for consideration of these conditions: several members characterised them as "monstrous"; the proposal to adopt an income limit especially rousing the ire of the meeting. After much discussion the secretary was directed to inform the Association that the conditions were impossible of acceptance. For a long time a war has been waging in New South Wales between the British Medical Association and the Australian Medical Association on the point of charges to members of Friendly Societies, culminating lately in a determined boycott by the British Medical Association of all other medical men. Recently a large deputation from the Australian Medical Association waited upon the Acting Chief Secretary of New South Wales (Mr. Flowers), with a complaint against the British Medical Association (which Association, it was pointed out, included only 600 of the 1,500 medical practitioners in New South Wales), and asked hm to appoint a Royal Commission of inquiry into the whole matter, to which he agreed.

### MUNICIPAL DENTISTS IN NEW YORK.

Medical inspection of school children is being taken up enthusiastically in New York. In the coming year £100,000 is to be spent on the children, numbering 700,000, who attend the municipal board schools, known locally as public schools. This sum is 33 per cent. more than was spent last year and will pay the cost of twenty-four municipal dentists. Seventy-two medical inspectors will examine the scholars at stated intervals and 196 nurses will visit the homes of the sick children to supervise their treatment. The teeth and the noses of the children are to receive elaborate attention, for the modern opinion is that to the disease of these organs is due the remarkable backwardness of the pupils. We sometimes wonder whether the Americans really carry out the vast plans that they advertise so assiduously, but our own problems at home remind us that medical inspection is not a thing to be played with. Our business is to see that lack of funds on this side does not prevent it from being carried out.

### REMARKABLE PROGRESS AT LEEDS.

A capital example of what enthusiasm and energy can achieve is seen in the astonishing progress of the King Edward Memorial Fund at Leeds. We recorded on February 4 last (page 561) that some £70,000 had been received as a part response towards the £150,000 which was asked for in order to reconstruct, and perhaps to remove, the whole infirmary building. Two-thirds of this great task has been accomplished, and the success of the appeal, together with the hard work which has helped to win it, are a monument of what can be done under the inspiration of the voluntary system. As our report on January 29, 1910, was one of the first steps in this direction, we are especially hearty in congratulating Mr. Lupton, Mr. W. Middlebrook, M.P., the Lord Mayor, and the people of Leeds. Two years ago this work was called impossible; to-day £110,000, or over two-thirds, have been raised. Let other cities remember Leeds when the old cry is raised, "We cannot get the money."

### A PATIENT'S GRATITUDE.

A young girl of the name of Ruby Watson, of Ryston End, Downham Market, Norfolk, was admitted into Addenbrooke's Hospital, Cambridge, very ill indeed some time last year. She left the hospital after a stay of nine months much improved but not cured. The hospital has just received an unexpected contribution in the form of a cheque for twelve guineas from this young patient as an expression of gratitude for the treatment she received during the nine months she was an in-patient. Miss Watson was so grateful for the kindness and attention she received at the hands of everyone in the hospital that, although a confirmed invalid practically, she felt she must do something for the hospital. Being somewhat skilful with her needle, she decided to work an autograph afternoon teacloth for the express purpose of raising funds for Addenbrooke's Hospital. Upon the cloth, which is a large one, Miss Watson worked in coloured silks the names of as many as were willing to contribute a small sum to the hospital. Each person affixed his or her name upon the white linen cloth in pencil, and then Miss Watson worked in his or her autograph in silk, thus the cloth has been covered with autographs representing many hours of patient labour. Miss Watson sent the cloth to the Secretary-Superintendent of the hospital with a letter in which she says: "I should very much like all the staff to see the cloth, and if it is convenient that it should be hung in the hospital so that if anyone of the visitors would care to undertake a similar manner of raising funds it would give them an idea." She adds, "I have done my very best to get as much as I can, and trust that the amount collected may be of service to the hospital authorities and patients. If I can find another way of doing something for Addenbrooke's I shall be delighted to do it later on. I might say I

have found great pleasure in doing a little to help the good work which is carried on at the hospital, and shall always feel ready to do any slight service I can." This clearly shows what can be accomplished by a single patient possessing a grateful heart. The idea of the cloth was suggested to Miss Watson by her sister, who had seen something like it done elsewhere. A similar cloth, it may be remembered, was worked some time ago by the Hon. Secretary of the Addenbrooke's Hospital Ladies' Needlework Guild (Mrs. Offord) in aid of the fund being raised for the re-establishment of children's wards at the hospital, and it realised £27.

### PROGRESS AT THE PONTYPOOL HOSPITAL.

The Pontypool and District Hospital, now in its ninth year, seems to be progressing steadily, and to be maintaining its financial position notwithstanding the fact that the present year has seen many important changes. First of all, the new Capel wing was opened in the early part of the year, and though it was impossible to publish a full statement of the cost at the half-yearly meeting in August, we believe that by this time the details are known. Another change was the appointment of a new secretary in the person of Mr. T. Morgan, to succeed Mr. W. H. Hughes, who resigned on taking up a new appointment, after having been connected with the institution from the first. This is eminently a working-men's hospital, and the financial year, we understand, is showing that Mr. Morgan is well maintaining the hospital's grip on their regard.

### A DAY'S WORK IN A HOSPITAL DISPENSARY.

Although this department is one of the most important in hospital administration, yet it is the one which is known less than any other by the people who are engaged in hospital service. This is probably due to the fact that the majority of the employees very seldom come into actual contact with the dispensers. It is surprising to find out how little is known of the actual work in the hospital dispensary. Many people imagine that the drugs, etc., which are used are bought ready for use from the manufacturing chemist and that the work consists principally of mixing a few liquids together in a bottle, filling boxes with ointments, and weighing a few powders. They quite overlook the fact that a great many preparations must be made before these simple operations can be performed, and it is certain that the majority would be surprised if they only knew how many important preparations are made each day by the pharmacist, with skill and care. Frequently it is necessary for the pharmacist to consult the prescriber regarding the mode to be adopted and occasionally to advise an entirely different form of dispensing some special drug, and it will be easily understood that these consultations are considered not the least important incidents in the day's work.

### THE WORRIES OF A HOSPITAL PHARMACIST.

The dispensing for the out-patients may be considered the most interesting, and this portion of the day is a very busy one. In the "O. P." department one finds all sorts and conditions of men and women, both native and foreign. A dispenser's experiences amongst this class of patients would require an article to itself, and be well worth reading too. Apart from the ordinary work of the dispensary, the pharmacist must be an experienced man of business, devoting a great deal of time to the study of the drug and other markets, because a careful and observant man is often able to save money for his department by being able to ascertain the right time to buy.

### DISPENSERS UNDER THE INSURANCE BILL.

Last week a question was asked in Parliament by the member for Colchester, who desired to know whether a certified assistant to an apothecary under the Apothecaries Act, 1815, now acting as one of the dispensers at the Metropolitan Hospital, Kingsland Road, will be qualified under Section 14 (5) (3) of the National Insurance Bill as now amended; and, if not, whether, in view of the undertaking given by him, such further amendment will be proposed by the Government as may be necessary to prevent his exclusion. Mr. McKinnon Wood in reply said that such a person, if he has been acting for not less than three years, could be employed by a pharmacist in dispensing under the Bill, and that the Chancellor was prepared to consider whether any amendment was necessary on report. A question relating to the position of voluntary hospitals is asked by Mr. Samuel Roberts, who desires to be informed if the Chancellor has received a copy of a joint resolution passed by the boards of management of the four voluntary hospitals in the city of Sheffield, calling upon the Government so to amend the National Insurance Bill that the continued existence of voluntary hospitals may be safeguarded financially and their efficiency as curative institutions and schools of medicine may be maintained and developed; and whether he proposes to take any, and what, steps to safeguard the interests concerned.

### THIS WEEK'S DRUG MARKET.

Business in the drug market has been quieter, and few articles have attracted special attention. Menthol has ceased to advance in price, the tendency being rather in the other direction; the article is obtainable at a few shillings per pound less than was quoted a week ago, and further reductions are not unlikely to take place before long. There has again been a good demand for eucalyptus oil, but there has been no further advance in price. Cod-liver oil also continues in good request, the price tendency still being in the downward direction. Oil of aniseed is again slightly dearer; oil of cloves is somewhat lower in value. The prices of opium, morphine, codeine, ergot of rye, sugar of milk, santonin, and hydrastis canadensis are well maintained. Castor oil is slightly dearer. Cream of tartar is a shade cheaper and buchu leaves are fetching rather higher prices.

